# More than Ninety Percent of the Light Energy Emitted by Near-Infrared Laser Therapy Devices Used to Treat Musculoskeletal Disorders Is Absorbed within the First Ten Millimeters of Biological Tissue

**DOI:** 10.3390/biomedicines10123204

**Published:** 2022-12-09

**Authors:** Leon Kaub, Christoph Schmitz

**Affiliations:** Department of Anatomy II, Faculty of Medicine, LMU Munich, 80331 Munich, Germany

**Keywords:** laser therapy, musculoskeletal system, tissue penetration depth, laser beam characterization

## Abstract

There is increasing interest in the application of near-infrared (NIR) laser light for the treatment of various musculoskeletal disorders. The present study thoroughly examined the physical characteristics of laser beams from two different laser therapy devices that are commercially available for the treatment of musculoskeletal disorders. Then, these laser beams were used to measure the penetration depth in various biological tissues from different animal species. The key result of the present study was the finding that for all investigated tissues, most of the initial light energy was lost in the first one to two millimeters, more than 90% of the light energy was absorbed within the first ten millimeters, and there was hardly any light energy left after 15–20 mm of tissue. Furthermore, the investigated laser therapy devices fundamentally differed in several laser beam parameters that can have an influence on how light is transmitted through tissue. Overall, the present study showed that a laser therapy device that is supposed to reach deep layers of tissue for treatments of musculoskeletal disorders should operate with a wavelength between 800 nm and 905 nm, a top-hat beam profile, and it should emit very short pulses with a large peak power.

## 1. Introduction

There is increasing interest in the application of near-infrared (NIR) light for the treatment of various musculoskeletal disorders, where the NIR light is used to reduce pain and inflammation and to stimulate tissue healing [1,2,3,4,5]. A number of studies showed that high power laser therapy alone can effectively be applied to treat various musculoskeletal disorders (for examples and references, see [6]).

The use of NIR laser light as a treatment modality is largely based on empirical results, and there is a general lack of understanding of the underlying molecular and cellular mechanisms of action [2,5,7]. On the other hand, there is growing evidence that in biological tissues, the energy of NIR laser light is predominantly absorbed by mitochondria (the organelles in the cells that generate most of the cell’s supply of adenosine triphosphate, which is used by the cells as a source of chemical energy) and the rough endoplasmic reticulum (the organelles in the cells where the protein biosynthesis takes place), and that the main modes of action take place at these intracellular structures [8,9]. Furthermore, in a study investigating the analgesic effect of a pulsed laser with a wavelength of 904 nm (hereafter: pulsed 904 nm laser), it was shown that laser treatment led to a reduced firing rate of heat nociceptors in the tongue of anesthetized cats [9]. Another study showed a reduced prostaglandin E2 (PGE_2_) concentration in the paratenon after treating human Achilles tendinopathy with a pulsed 904 nm laser [10]; PGE_2_ plays a central role in inflammation and pain mediation [11]. These data indicate that subjects suffering from musculoskeletal disorders can benefit from treatment with NIR laser light.

Another potential application of NIR laser light in medicine is the augmentation of extracorporeal shock wave therapy (ESWT) [6]. ESWT is a non-pharmacological and non-surgical treatment modality for a variety of musculoskeletal disorders [12]. The molecular and cellular mechanisms of action of extracorporeal shock waves (ESWs) on musculoskeletal tissue were addressed in more than 180 studies in the literature [13]. A key mechanism is the reduction of pain sensation and a blockade of neurogenic inflammation through the overstimulation of substance P nerve fibers [14]. Due to this action on substance P nerve fibers, ESWT can become very painful at high energy doses. On the other hand, two studies demonstrated that high energy doses may lead to a more efficient treatment. Specifically, an earlier study on the management of calcifying tendonitis of the shoulder with focused ESWs [15] and a recent study on the management of knee osteoarthritis with radial ESWs [16] demonstrated that application of a certain number of ESWs with high energy flux density (EFD) resulted in a clinically better outcome compared to the application of twice the number of ESWs with half of the initially used EFD [15,16]. Furthermore, it was demonstrated that the effect of ESWT on substance P nerve fibers may become blocked through local anesthesia [17,18]. This has led to the general recommendation not to use local anesthetics to increase the pain threshold during ESWT [12,18]. On the other hand, due to the aforementioned mechanisms of action, a pretreatment with a high power NIR laser may locally and temporarily reduce the sensation of pain and, therefore, allow the application of ESWs with higher EFD in subsequent ESWT [6]. Additionally, the combination of high power NIR laser treatment with ESWT may lead to synergistic effects [6].

While some musculoskeletal tissues are close underneath the subcutaneous layer of skin (e.g., the Achilles tendon), other target tissues are deep within the body (e.g., the origin of the hamstring muscles at the ischial tuberosity). It is, therefore, important to know the amount of energy that transmits into certain depths of tissue in order to better understand the effects of NIR laser light in the treatment of musculoskeletal disorders. The penetration depth of laser light in tissue depends on many parameters of the input laser beam, such as the wavelength and beam width [19,20], as well as on parameters of the investigated tissue such as the skin pigmentation [21].

Furthermore, there are different operating modes of a laser. Commercially available laser therapy devices often offer continuous wave (CW) laser light and/or pulsed laser light. The latter is generally believed to lead to better clinical results compared to CW laser light [22], since it can deliver higher peak powers into deep layers of tissue with less undesired surface heating [2], and it more efficiently targets small intracellular structures such as mitochondria [8]. However, the term pulsed laser light is sometimes unclear and parameters such as the pulse length, repetition rate and peak power are often not well reported, although they can differ greatly between different laser therapy devices [22].

A common way to measure the penetration depth of laser light into biological tissue is to measure the laser light power that gets transmitted through slices of tissue specimens with varying thicknesses (e.g., [23,24,25,26]). However, most studies that investigated the penetration depth in tissue did not fully characterize the used laser beams. Other studies compared the penetration depths of laser beams that had differences in several important laser beam characteristics, but assigned their results to a difference in only one of the parameters. For example, a recent study reported that 904 nm light penetrated deeper than 660 nm and 830 nm light [25]. However, the authors of this study compared a pulsed 904 nm laser beam with two CW laser beams of wavelength 660 nm and 830 nm. It is, therefore, unclear whether the differences reported in [25] resulted from the different wavelengths or from the different operating modes of the laser devices investigated in [25]. In addition, studies that reported penetration depth measurements often ignored that some part of the incoming light is reflected at the interface between air and tissue (e.g., [23,26,27]). These reflection losses are difficult to determine properly; however, they can be estimated during the computation of penetration depths.

The present study thoroughly examined the physical characteristics of laser beams from two different laser therapy devices that are commercially available for the treatment of musculoskeletal disorders. Then, these laser beams were used to measure the penetration depth in a broad range of biological tissues from different animal species, during which important laser beam parameters such as the beam width were kept constant. Reflection losses were considered when computing corrected penetration depths. Laser beam characteristics and penetration depths in biological tissues were investigated at the wavelength of 905 nm. In addition, one of the laser therapy devices was used to investigate the effects of different wavelengths on the penetration depth in biological tissues.

Specifically, the present study tested the hypotheses that (i) corrected penetration depths of pulsed NIR laser light in biological tissue are larger than uncorrected penetration depths that were reported in the literature (e.g., [23,26,27]), but (ii) in line with the results of these studies in the literature, the depth at which 90% of the light energy is absorbed is within the range of 5 mm to 10 mm. This would be of high relevance for treatments of musculoskeletal disorders using lasers, since it would indicate that pathologies at a depth of 10 mm and higher below the skin surface could only be reached effectively with short pulses of NIR laser light with a large peak power.

## 2. Materials and Methods

### 2.1. Biological Tissue Specimens

Penetration depths were measured for eight different biological tissue specimens, including skin, fat, muscle, tendon and bone tissue. The tissue specimens came from three different animals (chicken, pig and beef) and were freshly purchased in a butcher shop in Munich (Germany); thus, no ethics approval or registration of the present study with authorities was required. From each type of tissue, multiple slices with varying thicknesses were cut, ranging from 1.6 mm to 24 mm (Table S1). The thickness of the slices was determined before and after the measurements using a medical ultrasound device (HS-3000, Honda Electronics, Toyohashi, Japan); the mean of the two thickness measurements was used for data analysis. The thicker porcine skin specimens also contained fat and muscle tissue underneath the skin. Bone tissue specimens (porcine ribs) were not cut into slices. Instead, the laser light was either irradiating a part of the tissue specimen where bone was within the tissue, or the intercostal space in between two bones (mainly muscle tissue). The thickness of the bones within the tissue was 13 mm. During measurements, the tissue specimens were kept at room temperature. They were stored in plastic foil whenever they were not measured.

### 2.2. Instruments and Procedures

**Figure 1:**
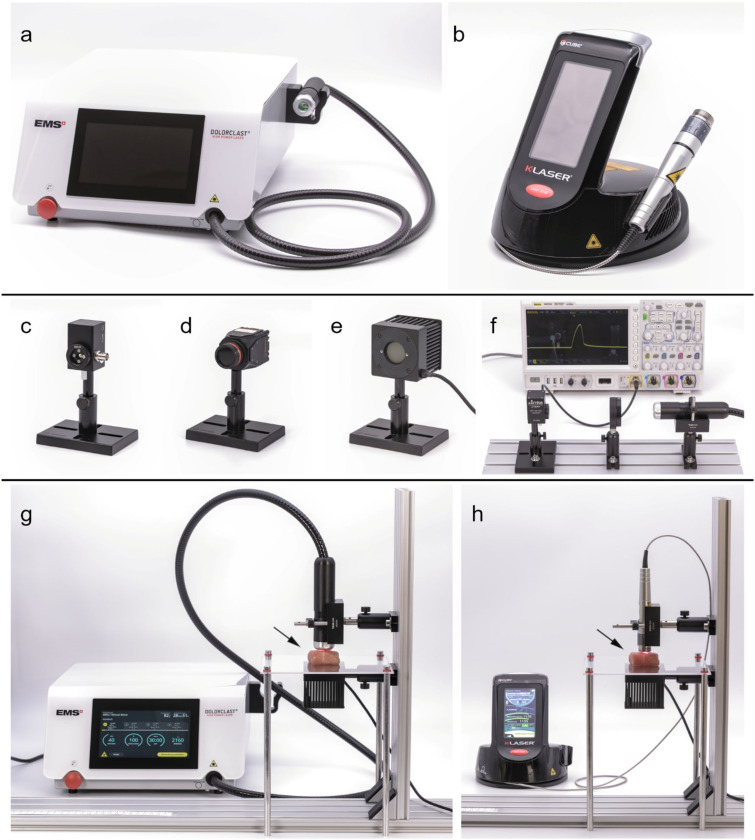
Laser therapy devices, sensors and experimental setups used in the present study: (**a**) EMS laser (Dolorclast High Power Laser; Electro Medical Systems, Nyon, Switzerland); (**b**) K-laser (Cube 4; Eltech K-Laser; Treviso, Italy); (**c**) Fast Photodiode (FPD-VIS-300; Ophir Spiricon Europe, Darmstadt, Germany) for temporal beam characterizations; (**d**) experimental setup for laser pulse characterization measurements with the handpiece of the EMS laser (right), the FPD (left) and an optical diffusor in the beam line (center); the FPD is connected to an oscilloscope (MSO7024; Rigol Technologies, Suzhou, China); (**e**) beam profiling camera (LT665; Ophir Spiricon Europe) that can measure spatial power density maps; (**f**) thermal power sensor (50(150)A-BB-26-PPS; Ophir Spiricon Europe); (**g**) experimental setup for penetration depth measurements with the EMS laser; (**h**) experimental setup for penetration depth measurements with the K-laser. The arrows in (**g**,**h**) point to a tissue specimen (15 mm thick chicken muscle tissue) on the specimen holder.

The following commercially available laser therapy devices were investigated and used for this study: (i) Dolorclast High Power Laser (Electro Medical Systems, Nyon, Switzerland) (hereafter: EMS laser) and (ii) Cube 4 (Eltech K-Laser, Treviso, Italy) (hereafter: K-laser). The EMS laser and the K-laser are shown in Figure 1a,b, with specifications provided by their manufacturers summarized in Table 1.

The laser beams of each laser therapy device were analyzed using three different sensors. Since both laser therapy devices were operated in pulsed wave (PW) mode throughout the experiments, the laser pulses were recorded in time with a fast photodetector (FPD) sensor (Figure 1c) (FPD-VIS-300; Ophir Spiricon Europe, Darmstadt, Germany). The FPD was connected to an oscilloscope (MSO7024; Rigol Technologies, Suzhou, China) to record measurements at a sampling rate of 10 Gigasamples per second. The sensor was protected from high power densities by placing an optical diffusor in the beam line (Figure 1d). In addition, power density maps were recorded using a laser beam profiling camera (LT665, Ophir Spiricon Europe) (Figure 1e). Two factors were computed from power density maps to analyze the beam profiles that are both according to ISO standards [28]: the roughness of fit factor and the plateau uniformity. The roughness of fit shows how well a measured distribution fits to a given theoretical distribution, with zero being a perfect fit. The plateau uniformity analyzes how close a distribution comes to an ideal top-hat distribution. A top-hat beam corresponds to a laser beam with uniform power density within a circular disk around the center of the beam. The closer the value of the plateau uniformity comes to zero, the flatter is the beam profile in its center. The camera was operated with software from the manufacturer (BeamGage Professional v6.17.1; Ophir Spiricon Europe). Power measurements were performed with a thermal power sensor (Figure 1f) (Model 50(150)A-BB-26-PPS; Ophir Spiricon Europe) which had a noise level of 2 mW. The power sensor came with the ability to measure beam widths to an accuracy of 0.1 mm.

To measure the penetration depth of the laser light in the investigated tissues, the tissue specimens were placed on an acrylic glass plate (thickness, 2 mm). The laser beams irradiated the tissue specimens from above, and the thermal power sensor was placed below the specimen holder to measure transmitted power. The experimental setup for the EMS laser is shown in Figure 1g, and for the K-laser in Figure 1h. This setup was similar to the procedures used in other studies [23,25,26,27].

All power measurements were normalized to the according beam widths, leading to values of power densities. Power density measurements were further normalized to the power density of the laser beam irradiating the empty specimen holder. Power and beam sizes of the laser beams, therefore, were measured before each tissue specimen was placed on the specimen holder. Once a tissue specimen was in between the laser emitter and the power sensor, beam sizes were often larger than the aperture of the sensor due to scattering in the tissue. In that case, power densities were computed using the full aperture of the sensor (26 mm) as the beam width. Since the tissue specimens were not perfectly flat, each tissue specimen was measured seven times with the laser light irradiating slightly different locations on the tissue specimen’s surface, and power measurements were averaged. Beam size measurements were also used to adjust the incident beams of both laser therapy devices to the same diameter (approximately 15 mm). This adjustment was necessary since penetration depths can strongly depend on the size of the incident beam [20].

Additionally, temporal profiles of the pulsed laser beams that were transmitted through tissue specimens were measured by placing the FPD underneath the tissue specimen holder. This was performed for porcine skin specimens with thicknesses ranging from 4.2–21.4 mm. Besides a different sensor, the experiment was performed in the same way as described for the power sensor.

The EMS laser operated at a wavelength of 905 nm, while the K-laser could be used at four different wavelengths: 660 nm, 800 nm, 905 nm and 970 nm. In addition, the K-laser had a so-called intense super-pulse (ISP) mode, which used all four wavelengths. In the present study the K-laser was mainly operated at 905 nm to be able to compare the results to the EMS laser. The other wavelengths of the K-laser and its ISP mode were additionally analyzed for selected tissue types. Of note, the 660 nm beam of the K-laser had a maximum power of 100 mW and, therefore, was not used in any of the experiments. All penetration depth measurements were performed using the laser therapy devices in PW mode at their maximum repetition rates (EMS laser, 40 kHz; K-laser, 20 kHz) and with an average power of 1–1.2 W. The K-laser in ISP mode set to 1 W, resulted in 1.8 W measured average power. Since all measurements were normalized to the incoming average power, small differences in average power were tolerated. Power measurements were stable 90 s after the laser emitters were switched on (Figure S1). Therefore, all experiments were started no earlier than two minutes after switching on the laser emitters.

### 2.3. Penetration Depth Analysis

The penetration depth can be computed from the above-described measurements using the Beer–Lambert law. When measuring the incident power density $P_{i}$ and the transmitted power density $P_{t}$, one can compute the transmittance within the tissue $T_{t}$ as

(1) $$T_{t}=\frac{P_{i}}{P_{t}\left(d\right)}=e^{-\mu d}$$

with the tissue thickness d and the absorption coefficient μ. Equation (1) describes the exponential decay of power that is due to absorption within the tissue. It does not take other power losses such as reflection losses into account. Since for the present study the laser emitter and the sensor were outside of the tissue, there were two air–tissue interfaces in the experimental setup with potential reflection losses. For each of these interfaces, the decay in Equation (1) can be multiplied with the factor 1−R, with R being the reflectance [24,25]. Since R is symmetric, it is the same for both the air–tissue interface where the laser beam enters the tissue specimen and the air–tissue interface where the laser beam leaves the tissue specimen, and the transmittance becomes:

(2) $$T=\frac{P_{i}}{P_{t}\left(d\right)}={\left(1-R\right)}^{2}e^{-\mu d}$$

When fitting the function $f=ae^{-bd}$ to the measurements, it can be seen from Equation (2) that the best fitting parameters will correspond to $a={\left(1-R\right)}^{2}$ and b=μ. By taking the square root of the parameter a, a corrected penetration depth can be computed in which one of the interfaces is still considered in the computations. This was accepted, as there is also such an interface during medical treatments with a laser therapy device (where the laser beam enters the skin). The presented correction method can only be an approximation of the transmittance and penetration depths. However, it comes with the advantage that it avoids the need of including external parameters. Such parameters (e.g., refractive indices) are often highly variable and depend on the individual experimental setup of a given study. A recent study with a comparable study design presented a similar method to consider reflection losses [25]. However, the method used in [25] requires knowledge of the refractive index of the investigated tissue, which in [25] was taken from the literature. The correction method of the present study avoids the necessity to estimate refractive indices.

Of note, another recent study [29] concluded that the simple form of the Beer–Lambert law that was used in the present study fails at high absorber or scatterer concentrations, but in combination with a correction for reflection losses (as performed in the present study) it gives reliable results for biological tissues such as the specimens used in the present study [29].

In a number of studies in the literature the penetration depth was defined as the thickness at which $P_{t}$ was decreased to 1/e of $P_{i}$ [24,26,30,31]. However, in the present study, the 10% and 1% penetration depths were computed since these tend to better show differences between the investigated laser therapy devices and tissue types. Curve fittings were performed with the method of least-squares using software that was self-written in the open source programming language Python (version 3.9, Python Software Foundation, Delaware, MD, USA).

## 3. Results

### 3.1. Laser Beam Characterization

**Figure 2:**
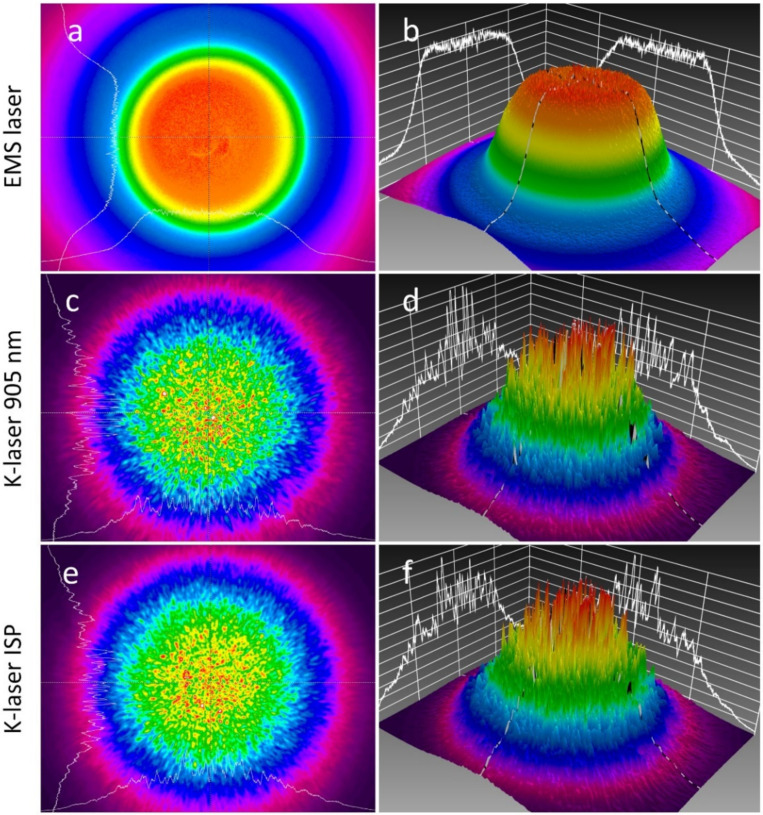
Power density maps recorded with a beam profiling camera for the EMS laser (**a**,**b**), the K-laser using 905 nm wavelength (**c**,**d**), and the K-laser operated in ISP mode (**e**,**f**). Two-dimensional power density maps (**a**,**c**,**e**) are shown as well as three-dimensional representations (**b**,**d**,**f**). White lines in both representations are beam profiles along the x- and y-axis. Maps were normalized to the maximum intensity of each recording.

The laser beams emitted by the EMS laser and the K-laser had fundamentally different properties in the spatial and temporal domains. Power density maps recorded with the laser beam profiling camera showed that the EMS laser had a relatively flat power distribution in the center area, surrounded by a sharp power decay. This type of distribution is typically called a top-hat distribution. For the EMS laser, the roughness of fit was 0.25 and the plateau uniformity was 0.08, proving that its beam came close to a perfect top-hat distribution (Figure 2a,b). In contrast, the K-laser had more Gaussian-like distributions and much larger noise levels. The roughness of fit was 0.51 (905 nm) and 0.45 (ISP mode); it did not follow a top-hat distribution as the plateau uniformity was 0.50 (905 nm) and 0.75 (ISP mode). Differences in these values between the 905 nm diode and the ISP mode of the K-laser were small, and the similarity between these can also directly be seen in the power density maps (Figure 2c–f).

**Figure 3:**
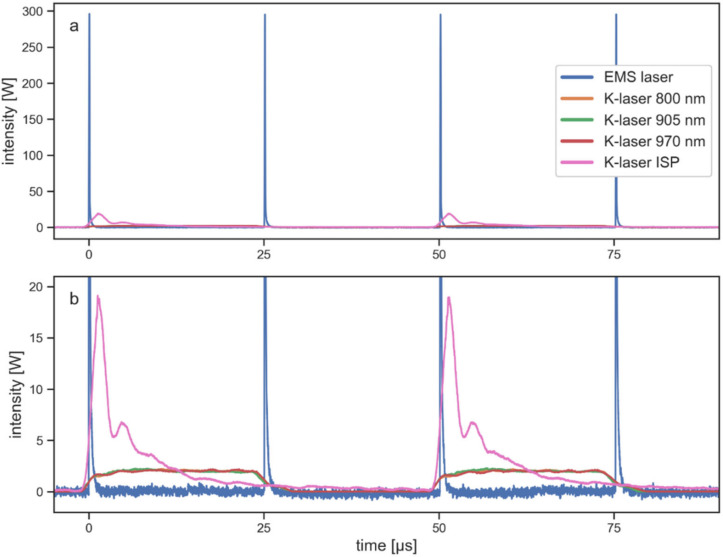
Light intensity of the two laser therapy devices investigated in the present study in time recorded with the fast photodetector sensor (FPD). The same signals are plotted showing the full range (**a**) and using a smaller range to better depict the signals of the K-laser (**b**). Since the FPD is recording relative measurements, intensity values were scaled to fit the power that was used during the penetration depth experiments. Peak power was 300 W for the EMS laser, 20 W for the K-laser in ISP mode and 2 W for the individual diodes of the K-laser. Average powers of all laser beams were set to 1–1.2 W. Repetition rates were set to the maximum of each device: 40 kHz for the EMS laser and 20 kHz for the K-laser. A moving-average filter was applied to the signals; the observed differences in noise levels were mainly introduced by scaling.

Differences between the laser therapy devices and their emitted beams were also visible in temporal profiles, i.e., pulse shapes. All signals were recorded with the laser therapy devices set to a similar average power of 1–1.2 W. However, the peak powers and pulse lengths of the laser beams were fundamentally different. The peak power of the EMS laser was approximately 15 times larger than the peak power of the K-laser operated in ISP mode, and about 150 times larger than the peak power of the individual wavelengths of the K-laser. The EMS laser could reach such high peak power due to its very short pulse lengths of approximately 100 ns. In contrast, the pulse length of the individual wavelengths of the K-laser was 25 µs. As it can be seen in Figure 3, the laser beams generated by the K-laser in non-ISP mode emitted constant light during a half cycle and no light for the remaining cycle, i.e., they emitted light for 50% of the time.

The pulse length of 25 µs of the K-laser, therefore, was set by the repetition rate of 20 kHz. This type of pulsing was fundamentally different from the pulsing of the EMS laser, which always emitted the same pulses regardless of the repetition rate. With a repetition rate of 40 kHz, the EMS laser emitted light during 0.4% of the time. The K-laser operated in ISP mode emitted pulses with a different shape than the pulses of the non-ISP modes. Similar to the EMS laser, the pulse length of the ISP pulses was independent of the repetition rate. However, the present study found that for this mode an increase in average power led to an increase in pulse length and not in a change of peak power or repetition rate (Figure S2). At the maximum available average power, the pulse length of the ISP mode signal was 25 µs and, thus, exactly the same as the pulse lengths of the K-laser’s non-ISP signals. The signal for the ISP mode shown in Figure 3 was measured with the average power set to 1 W. With this setting, the pulse length of the first main pulse was approximately 2.5 µs. As it can be seen in Figure 3, the pulses of the K-laser’s ISP mode had an initial main pulse followed by a relatively long decay. Therefore, the actual pulse length was larger than 2.5 µs. This 1 W signal was the one used for the penetration depth measurements.

### 3.2. Penetration Depth of 905 nm Laser Light in Tissue

Penetration depths were measured for different tissue specimens using both laser therapy devices. Since the EMS laser operated at 905 nm wavelength, the K-laser was used at the same wavelength for comparative purposes. Both laser therapy devices were also set to the same average power and beam diameter. However, they differed in repetition rate and, as shown in the characterization of the laser beams (Figure 2 and Figure 3), had different peak powers, pulse lengths and beam profiles.

**Figure 4:**
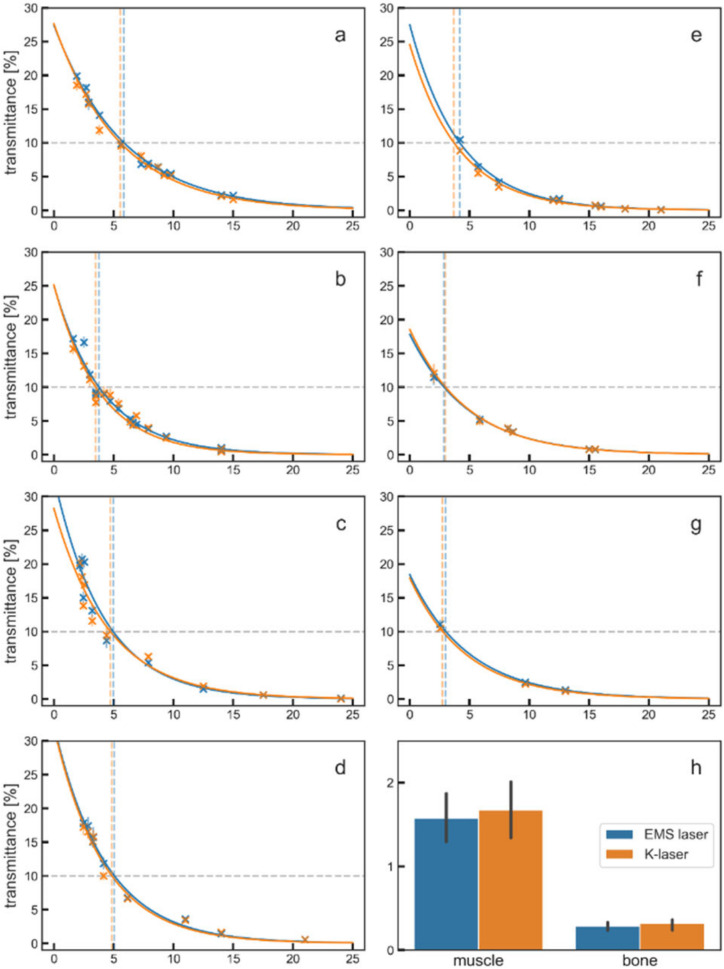
Results of penetration depth measurements for different tissues ((**a**), chicken muscle; (**b**), porcine muscle; (**c**), beef muscle 1; (**d**), beef muscle 2; (**e**), porcine skin; (**f**), beef fat; (**g**), beef tendon; (**h**), porcine muscle and bone). Transmittance in percent is plotted against the tissue thickness in millimeters for the EMS laser (blue dots and blue lines) and the K-laser (orange dots and orange lines) (**a**–**g**). In addition, bone tissue and muscle tissue were compared for a fixed thickness of 15 mm (**h**). Measurements were performed with muscle tissue specimens (**a**–**d**) and with non-muscular tissue specimens (**e**–**g**) from different animal species. Each point represents the average of seven measurements; the according standard deviations are indicated by vertical lines. Solid lines represent best fitting transmittance curves. The 10% penetration depth is illustrated by dashed vertical lines in the color of the according laser therapy device.

All transmittance curves followed an exponential decay as it was predicted from the Beer–Lambert law. The decay can be assigned to absorption and scattering of the laser light within the tissue. For all tissues, most of the initial power was lost in the first 1–2 mm, and there was hardly any power left after 15–20 mm of tissue. The laser beams from the two laser therapy devices resulted in very similar transmittance curves for all tissue types (Figure 4). Best-fitting transmittance curves were computed for all transmittance measurements, and the fitting quality was verified through the standard deviation of the residuals (Figure S3), which was below 1% for most tissues except for the porcine muscle tissue (EMS laser, 1.02%; K-laser, 1.07%) and the beef muscle 1 tissue (EMS laser, 1.89%; K-laser, 1.66%).

Bone tissue absorbed more laser light than muscle tissue. While approximately 1.5% of the initial power transmitted through muscle tissue of 15 mm thickness, only 0.25% of the same power was able to transmit through tissue of the same thickness that had a 13 mm bone inside. A difference between these tissues was expected, since bone tissue has a greater density than muscle tissue. The results however show that almost all of the initial light power did not travel through 13 mm thick bone tissue.

**Figure 5:**
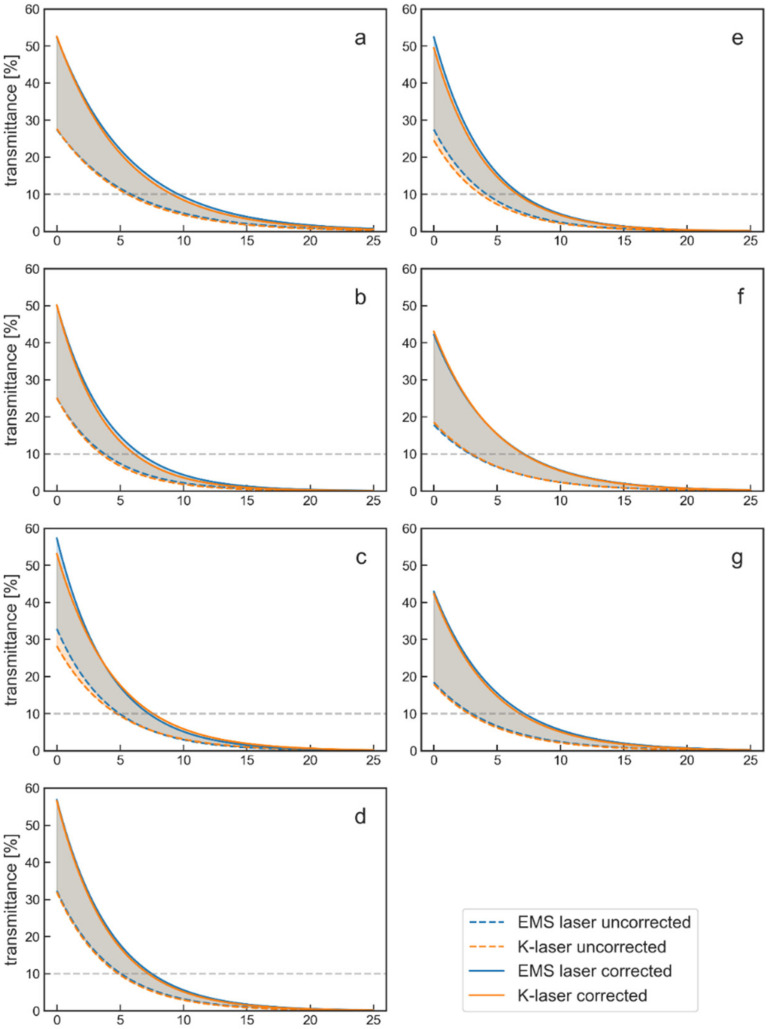
Transmittance curves before and after correction for different tissues ((**a**), chicken muscle; (**b**), porcine muscle; (**c**), beef muscle 1; (**d**), beef muscle 2; (**e**), porcine skin; (**f**), beef fat; (**g**), beef tendon). The transmittance in percent is plotted against the tissue thickness in millimeters. While the uncorrected curves are the same as in Figure 4, the corrected curves were added here as solid lines. The areas between the uncorrected and corrected curves are filled with the according color of the laser therapy device. This area gives the range of what are most likely the true transmittance values. Dotted gray lines represent the 10% penetration depths.

Based on the best fitting transmittance curves from Figure 4, corrected transmittance curves and penetration depths were computed. The corrected transmittance curves still included reflection losses from one of the air–tissue interfaces, but were corrected for the second interface (air–tissue) that usually does not exist in medical treatments. The corrected transmittance curves as well as the raw, uncorrected curves are shown in Figure 5.

Since the correction method was an approximation, the true values were most likely in the range between the uncorrected and the corrected curves, which is indicated in Figure 5. The data from porcine bone tissue were not corrected since these tissue specimens were at constant thickness, and no transmittance curves were measured.

**Figure 6:**
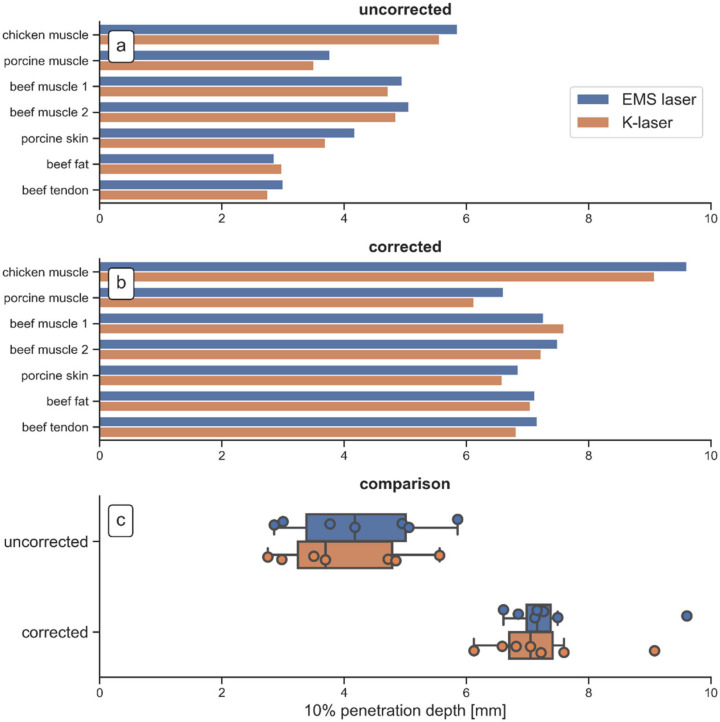
10% penetration depths of the EMS laser (blue bars) and the K-laser (orange bars) for all measured tissues: uncorrected penetration depth (**a**); penetration depth after correction for reflection losses (**b**); comparison of the uncorrected and the corrected 10% penetration depth (Tukey boxplots of all data shown in (**a**,**b**)) (**c**).

Based on the data shown in Figure 5, 10% penetration depths were computed for all measurements using the uncorrected and the corrected transmittance curves (Figure 6).

The uncorrected 10% penetration depths (Figure 6a) showed that the EMS laser penetrated slightly deeper into most tissues (six out of seven). Only for the beef fat tissue, the K-laser penetrated slightly deeper into the tissue. In the corrected data (Figure 6b), the EMS laser also penetrated slightly deeper into all investigated tissue specimens, except for one beef muscle tissue specimen. The larger penetration depth of the EMS laser was probably due to its higher laser beam qualities. Since both laser therapy devices were operated at the same wavelength, average power and beam diameter, differences in the penetration depth could have been caused by a better performance in one or several of the remaining laser beam parameters such as pulse length, peak power and repetition rate (Figure 3), or the beam profile (Figure 2).

Differences in penetration depth between the tissues were relatively small. The penetration in chicken muscle tissue was the deepest as seen in the uncorrected as well as in the corrected 10% penetration depths. The light travelling through the other investigated tissues showed relatively similar behavior, especially when looking at the corrected 10% penetration depths. The results from the two beef muscle measurements were highly similar, although they were performed on different days with fresh meat specimens on each day. Even the differences between the EMS laser and the K-laser were almost equal, proving the accuracy of the measurements reported in the present study. In general, the uncorrected 10% penetration depths showed higher variances than the corrected 10% penetration depths (Figure 6c). This can be explained by the differences in the reflectance of the tissues, since the correction method partly removed reflection losses. The corrected 10% penetration depths should, therefore, be more likely to represent true values. They showed that in most tissues, 90% of the initial laser power had decayed after approximately 7 mm of tissue.

Corrected penetration depth values for several thresholds (15%, 10%, 5%, 1%) are given in Table 2. Additionally, Table 2 shows transmittance values for different thicknesses (5 mm, 10 mm, 15 mm, 20 mm). These values correspond to the amount of power that remained at each tissue thickness.

### 3.3. Effect of Wavelength on Penetration Depth

The 800 nm and 905 nm light of the K-laser resulted in the highest penetration depths and in very similar transmittance curves. The 970 nm light of the K-laser clearly had the smallest penetration depth in all tissues. Light from the ISP mode resulted in intermediate penetration depths. This can be explained by the fact that the laser beam of the ISP mode was operating with a combination of all three individual wavelengths.

**Figure 7:**
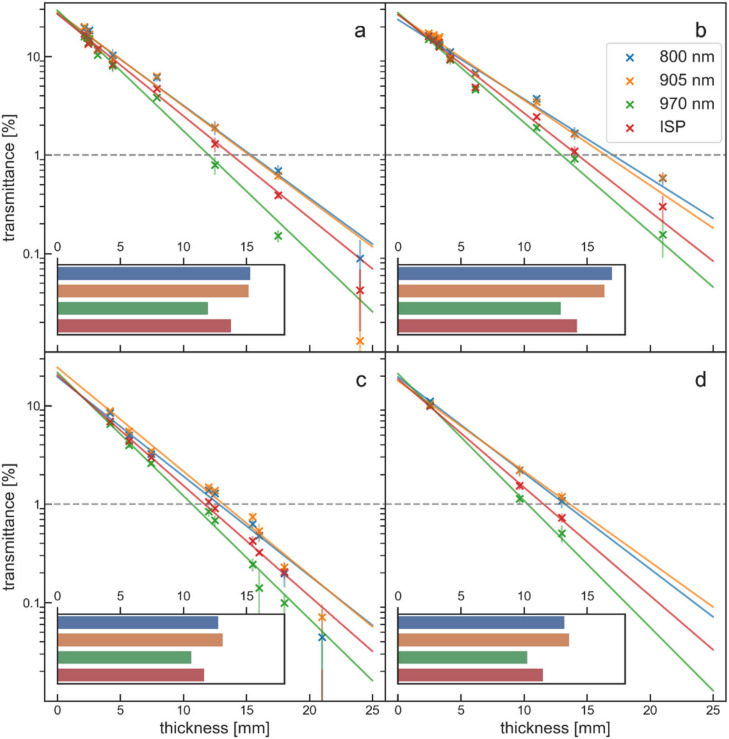
Transmittance of the three different wavelengths of the K-laser (800 nm (blue dots and blue lines), 905 nm (orange dots and orange lines), 970 nm (green dots and green lines)) and its ISP mode (red dots and red lines) for four different tissues ((**a**), beef muscle 1; (**b**), beef muscle 2; (**c**), porcine skin; (**d**), beef tendon). Each measurement is plotted with the according standard deviation. The uncorrected transmittance is plotted in logarithmic scale to enhance the difference between the different wavelengths and modes. The insets show for each tissue type the 1% penetration depths of the different K-laser wavelengths and modes as bar-plots. The insets have the same x-axis as the main penetration depth plots.

Figure 7 shows the transmittance curves from four different laser beams (800 nm, 905 nm, 970 nm, ISP mode) of the K-laser for four different tissues, without tissue-specific differences in the transmittance patterns.

### 3.4. Laser Pulses Transmitted through Tissue

The transmittance curves and penetration depths shown in Figure 4, Figure 5, Figure 6 and Figure 7 and Table 2 were measured with a power sensor. In addition, the FPD that measures temporal profiles of laser beams was used to investigate the penetration depth in tissue, using porcine skin specimens of varying thickness. The measurements of the FPD were normalized in two ways. In a first attempt all signals of one laser beam were normalized to the maximum of the signal that was transmitted through the thinnest porcine skin specimen (4.2 mm). Besides this, the signals were normalized to their individual maxima. Measurements of the empty specimen holder were not taken since these signals were too strong for the sensor.

**Figure 8:**
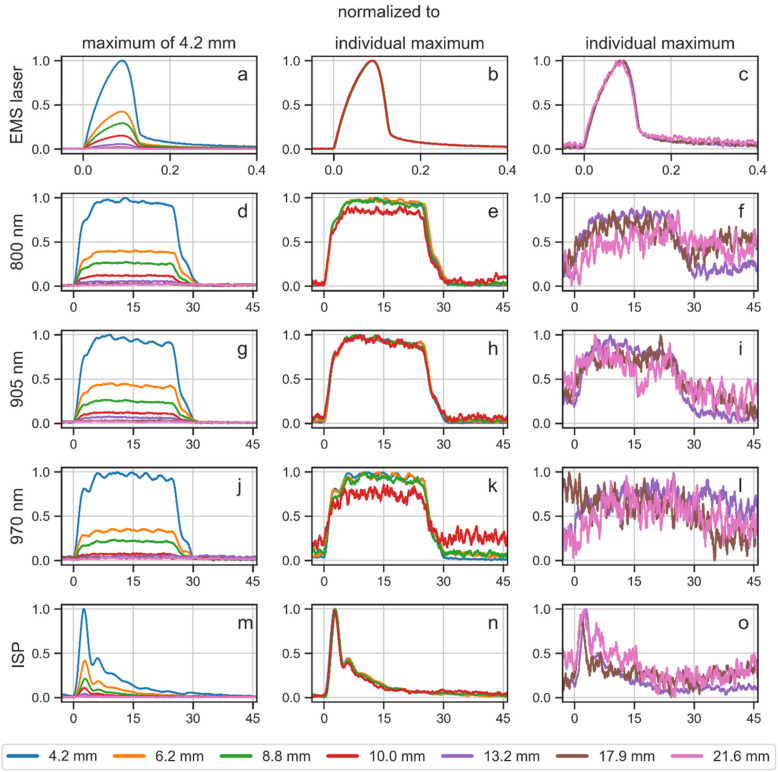
Laser beam pulses in time [µs] after transmission through porcine skin specimens of varying thickness: EMS laser (**a**–**c**) and the different modes of the K-laser (**d**–**o**). Pulse intensities were normalized to the maximum of the thinnest porcine skin specimen (**a**,**d**,**g**,**j**,**m**) or to their individual maxima (**b**,**c**,**e**,**f**,**h**,**i**,**k**,**l**,**n**,**o**), respectively. Since noise levels for thick tissue specimens were often large, pulses that were normalized to their individual maxima are shown in separate columns for thin tissue specimens (thickness between 4.2 mm and 10.0 mm) (**b**,**e**,**h**,**k**,**n**) and for thick tissue specimens (thickness between 13.2 mm and 21.6 mm) (**c**,**f**,**i**,**l**,**o**). Note that the x-axis of the EMS laser pulses spans 0.4 µs each (**a**–**c**), whereas the x-axis of the K-laser pulses spans 45 µs each (**d**–**o**). Accordingly, the laser pulses of the K-laser lasted on average more than 100 times longer than the laser pulses of the EMS laser.

Signals that were normalized to the maximum of the 4.2 mm thick porcine skin specimen showed that all recordings correlated well with the exponential decay seen in the power measurements. The signals that were normalized to their individual maximum demonstrated that the general pulse shape and the pulse length of all laser beams did not change with tissue specimen thickness (Figure 8).

Particularly for the EMS laser, it was found that even for the thickest porcine skin specimen (21.6 mm), there was a detectable signal that had the same pulse shape as the initial beam. The different wavelengths and modes of the K-laser also penetrated through most porcine skin specimens. However, these laser beams had more difficulties penetrating through tissue. The 970 nm wavelength beam was already deterred at 10 mm thickness and was barely detectable at 13.2 mm thickness. The 800 nm and 905 nm laser beams were transmitted through tissue better, although at thicker tissue specimens the signals had large noise levels. As in the power measurements, these two wavelengths showed similar behavior. The laser beam of the K-laser’s ISP mode was transmitted through porcine skin specimens up until 17.9 mm thickness, but the signal at the 21.6 mm tissue specimen was barely detectable.

## 4. Discussion

The key result of the present study was the finding that more than 90% of the light energy emitted by the investigated laser therapy devices was absorbed within the first ten millimeters of biological tissue. Thus, the hypotheses of the present study were confirmed. Other studies reported similar penetration depths of laser light in the order of several millimeters in biological tissues [24,30,32,33,34].

Of note, the investigations described in the present study could not be performed in humans in vivo. Such measurements would be invasive, and would require to cut certain organs (skin, subcutaneous fat, muscle) in vivo into slices of different thickness. Theoretically, it would be possible to carry out corresponding studies on laboratory animals in vivo. However, besides serious ethical concerns one could not exclude that the uninterrupted flow of blood in vivo could negatively impact the measurements and even destroy the highly sensitive measuring instruments. Thus, in the present study transmittances were measured for seven different tissues ex vivo from three different animal species, and most penetration depths were found to be very similar, especially after correcting for reflection losses (Figure 6). Only chicken muscle tissue showed larger penetration depths. Since especially the properties of porcine tissue are similar to the properties of human tissue [35], it is reasonable to hypothesize that the 10% penetration depth of light emitted by the investigated laser therapy devices in human tissue is also on the order of seven millimeters.

There are many factors that influence the penetration depth of NIR laser light in biological tissue, such as the laser beam characteristics, tissue specimen preparation and data analysis. These factors differed greatly among studies, making a comparison of the exact values of penetration depth difficult. Several studies used experimental setups similar to the ones used in the present study; however, most studies did not consider reflection losses when analyzing penetration depths [23,26,27]. In one study, the results were corrected for reflection losses; however, this study only reported absorption coefficients and no penetration depths [25]. Taken the mean absorption coefficients for 904 nm laser light reported in [25], 10% penetration depths in [25] can be computed to be 4.2 mm (porcine skin), 5.5 mm (porcine muscle) and 7.2 mm (porcine fat). These values are similar to the results of the present study, although large differences between muscle and skin tissue were not observed in the present study. A possible explanation for this difference might be the skin pigmentation, which has a strong influence on penetration depth [21]; the porcine skin specimens that were investigated in the present study had a very light skin color. Another study that took reflection losses into account measured penetration depths with a thermocouple array instead of a power sensor and operated a 1064 nm laser [24]. The results of this study [24], therefore, cannot be directly compared to the results of the present study. On the other hand, the penetration depths reported in [24] were also similar to the results of the present study (10% penetration depth for porcine muscle, 8.1 mm in [24]).

The method to correct transmittances for reflection losses applied in the present study can only be an estimation of the true penetration depth, since no reflectance measurements of the investigated tissue specimens were performed. Nevertheless, the correction resulted in more realistic values than ignoring reflection at all, and the similarity of the penetration depths reported in the present study to the results of other studies showed that the correction resulted in a realistic estimation of the penetration depth. In addition to the different data analyses, comparing values of penetration depth from different studies is challenging since there are many laser beam parameters that are affecting the penetration depth and, often, not all of these parameters were reported. Therefore, in the present study extensive temporal and spatial characteristics of the laser beams that were used for penetration depth measurements were included.

The two laser therapy devices investigated in the present study are both designed to be used for treatments of musculoskeletal disorders. However, they fundamentally differed in several laser beam parameters that can have an influence on how light is transmitted through tissue. In the present study, many of these parameters were kept constant in the penetration depth measurements in order to be able to compare the two laser therapy devices. Specifically, both laser therapy devices were operated at the same wavelength, beam size and initial power. While the wavelength and beam size are known to strongly affect the penetration of the emitted laser light in tissue [20], the initial power should not influence the penetration depth [36].

One of the largest differences between the two investigated laser therapy devices was their different way of pulsing the laser beam. In general, pulsed laser light has the advantage that it generates less tissue heating at the surface layer, because there are periods with no light power following each laser pulse [22,37]. This gives the tissue time to thermally relax and allows much higher peak powers during a pulse than the power of CW laser light. The EMS laser operated with very short pulses with a high peak power that was 250 times larger than its average power, and long periods in between two pulses. The K-laser on the other hand had a peak power that was only twice its average power and pulses that had the same length as the no-light periods (Figure 3). Since the K-laser’s way of pulsing is more similar to CW light, it results in more heating of the surface layer of tissue than the EMS laser. This is the case while both laser therapy devices delivered equal average powers into the tissue as seen in the penetration depth values. The present study also showed that the pulse lengths stayed constant when the laser light was transmitted through tissue (Figure 8). This implies that the peak power of the EMS laser at a given depth was still 150 times larger than the peak power of the K-laser. As an example, at 20 mm depth, the average power for both lasers was approximately 0.5% of the initial power. Therefore, the peak power of the EMS laser at this depth was still 1.5 W, while the peak power of the K-laser was 0.01 W. In addition, at a thickness of 21.6 mm, the pulse signal of the K-laser was barely detectable compared to a well-defined signal of the EMS laser (Figure 8). This shows that very short laser pulses with high peak power are more effectively transporting laser power into deeper layers of tissue. To our knowledge, this is the first study to report temporal profiles of laser light as it was transmitted through tissue.

Pulsed laser light seems to also result in more effective treatments than CW laser light [22]. A study on the effects of laser light in induced inflammatory processes found that a 905 nm pulsed laser resulted in a stronger inflammatory response compared to a 905 nm CW laser [8]. In this study [8], the pulsed laser (pulse length, 200 ns; repetition rate, 10 kHz; peak power density, 50 W/cm^2^) was operated with the same energy density as the CW laser. The authors argued that the pulsed light resulted in localized thermal activation of mitochondria and the rough endoplasmic reticulum, since these structures are in the correct size range to be target structures for a pulsed laser with length and repetition rate as used in [8]. Mitochondria and the rough endoplasmic reticulum also contain high relative amounts of lipid membranes that have an absorption peak at 930 nm [8]. Following this argument, not all pulsed lasers could achieve such effects, since pulse lengths that are longer than several hundred nanoseconds would not selectively target the same intracellular structures. From the laser beams investigated in the present study, the EMS laser had temporal characteristics (pulse length, 100 ns; repetition rate, 40 kHz) that could result in a similar selective photothermolysis as described in [8], while the pulse lengths of the K-laser beams were much longer (pulse length normal mode, 25 µs (normal mode) and 2.5 µs (ISP mode)). More studies about the intracellular target structures of differently pulsed laser light are needed in order to better understand the effect of pulsed laser light in biological tissue.

Besides the larger peak power, one study reported that pulsed laser light also penetrated deeper into tissue than CW light [38]. However, it was shown that these results were due to insufficient warm-up periods of the used laser device [39]. In the present study, therefore, it was assured that the investigated laser therapy devices emitted stable laser light by waiting a minimum of two minutes before starting any measurements to let the devices warm up sufficiently (Figure S1).

The two investigated laser therapy devices also showed differences in their spatial beam profiles. The EMS laser had a top-hat beam profile whereas the K-laser had a more Gaussian beam profile (Figure 2). The spatial profile of a laser beam does not affect the penetration depth directly; however, there can be significant differences in the local volumetric dosages [40]. The laser beam of the K-laser had the maximum power density in a relatively small area in its center, which can lead to an intensity that is much larger than the average power. In addition, the K-laser showed large noise levels and, therefore, uneven power distribution (Figure 2). The combination of a Gaussian profile and uneven power distribution can result in small spots with extraordinarily large power densities that can potentially lead to local irritation or even burning of skin. In contrast, a top-hat profile such as the profile of the EMS laser irradiates the skin evenly over the diameter of the beam, which leads to a uniform delivery of power into the tissue. Although an initial top-hat profile will transform into a Gaussian profile within the tissue due to light scattering [23], the advantage of being less likely to irritate skin makes a top-hat profile the preferred profile. The penetration depth is not directly affected by the spatial beam profile; however, it is strongly dependent on the beam size [19,20]. The beam size is a parameter that was often not reported or not considered in the data interpretation of other studies, which was criticized in the literature [22]. Therefore, in the present study, the laser beam size was kept constant for all experiments, all power measurements were normalized to the beam area, and all data analyses were performed with power densities.

The wavelength of a laser beam has a strong influence on the penetration depth in tissue (Figure 7). In the experiments performed in the present study, the 970 nm laser beam of the K-laser had a significantly higher absorption and, therefore, shorter penetration depth than the 800 nm and 905 nm laser beams. The poor performance of the 970 nm laser beam is in agreement with an earlier study that showed that 808 nm laser light penetrated significantly deeper into tissue than 980 nm laser light [23]. This effect is due to an absorption band of water at 976 nm [31,41], while 800 nm and 905 nm are in between the absorption bands for deoxyhemoglobin at lower wavelengths and water at higher wavelengths [20,42]. Since water is ubiquitous in all tissue layers, the 970 nm laser beam is mainly absorbed in surface layers of the tissue, resulting in an increase of the surface temperature. It has been reported that 810 nm CW laser light penetrated deeper into tissue than 904 nm pulsed laser light [39]. In contrast, another study reported that 904 nm laser light penetrated deeper than 830 nm laser light in four out of four tissues [25]. However, the 904 nm laser used in [25] had a larger laser beam size (diameter, 0.51 cm) than the used 830 nm laser beam (diameter, 0.31 cm), which could explain the reported difference in [25]. Based on measurements of the absorption and scattering spectra of tissue, the penetration depth of 800 nm and 905 nm light should be equal [31]. The absorption coefficient for 800 nm laser light is slightly smaller than for 905 nm laser light; however, this is to some extent compensated by a lower scattering coefficient at 905 nm [42,43]. For four different tissue types, no consistent difference between 800 nm and 905 nm laser light were found in the 1% penetration depths. Therefore, it was concluded that these two wavelengths penetrate equally well into tissue.

When operated in ISP mode the K-laser emitted shorter laser pulses and higher peak power than the pulses emitted in normal operation mode (Figure 3). Apparently, the ISP mode combined laser light from all three wavelengths of the K-laser, which may explain the intermediate penetration depth results (Figure 7). It seems like it would be more efficient if the K-laser dismissed the 970 nm diode for this mode, since light at this wavelength is mainly heating up water in the tissue. When operating the K-laser, it was observed in the present study that the peak power of the ISP mode was locked at 20 W. Therefore, at larger average powers, the K-laser operated with increased pulse lengths rather than with increased peak power and/or increased repetition rates (Figure S2). At the maximum average power, the pulses of the ISP mode had the same pulse length as the K-laser in normal mode. As discussed above, long pulses mainly result in tissue heating of the surface layers. Therefore, the ISP mode of the K-laser loses its advantage over the normal mode when used at larger average powers. The EMS laser, in contrast, increased the repetition rate when used at larger average powers, while the pulse length and peak power stayed constant. This seems to be beneficial since it avoids potential power losses at surface layers.

Overall, the present study shows that a laser therapy device that is supposed to reach deep layers of tissue for treatments of musculoskeletal disorders should operate with a wavelength between 800 nm and 905 nm, a large beam size and a top-hat beam profile, and it should emit very short pulses with a large peak power.

## 5. Conclusions

Analysis of the laser beams of two commercially available laser therapy devices for the treatment of musculoskeletal disorders (EMS laser and K-laser) revealed that they greatly differed in several beam characteristics. The laser therapy devices were operated with pulsed beams and the largest differences were seen in the parameters that define these pulses. The EMS laser emitted ultra-short pulses with large peak powers while the K-laser produced laser beams that were significantly longer and with much smaller peak powers. In addition, the laser beam of the EMS laser had a top-hat beam profile while the K-laser’s laser beams had Gaussian profiles with high noise levels. The penetration depths investigated with both devices at the same wavelength (905 nm), same beam width (15 mm) and same input power (approx. 1 W) were relatively similar. However, the EMS laser was capable of transmitting much larger peak powers than the K-laser due to its general pulsing mode. This shows that the laser beam characteristics are highly important for the penetration depth of laser light in tissue. Laser beam characteristics were often not well-reported in other studies or by the manufacturers of laser therapy devices. The wavelength also plays an important role for the penetration depth of a laser beam. It was found that 800 nm and 905 nm laser beams penetrated equally into tissue, while the 970 nm laser beam consistently reached less tissue depth. A combination of these three wavelengths was not as good as the 800 nm or the 905 nm beam alone due to the weak performance of the 970 nm beam.

Overall, this study presents estimates for the penetration depth of high power NIR lasers into biological tissues and shows which laser beam parameters are important to reach deep layers of tissue. This will help further studies about the effect of high power NIR lasers in the treatment of musculoskeletal disorders to determine the amount of energy that a laser therapy device can deliver to the target tissue.

## Figures and Tables

**Table 1:** Specifications of the two laser therapy devices investigated in the present study. Average power ($P_{average}$), peak power ($P_{peak})$, pulse length and repetition rate were provided by the manufacturers.

Laser Therapy Device	EMS Laser	K-Laser
Wavelengths [nm]	905	800/905/970
Modes	PW	CW/PW/ISP
$P_{average}$ [W]	1.2	12
$P_{peak}$ [W]	300	20
Pulse lengths	100 ns	25 µs–0.5 s
Repetition rates [Hz]	5–40 k	1–20 k

Abbreviations: nm, nanometer; W, Watt; Hz, Hertz; PW, pulsed wave; CW, continuous wave; ISP, intense super-pulse.

**Table 2:** Corrected penetration depths in millimeters for given thresholds, and corrected transmittance in percent at given tissue thicknesses.

		Corrected Penetration Depth [mm] for				Corrected Transmittance [%] at			
Laser	Tissue	15%	10%	5%	1%	5 mm	10 mm	15 mm	**20 mm**
EMS laser	Chicken muscle	7.3	9.6	13.6	23.0	22.1	9.3	3.9	1.7
	Porcine muscle	4.9	6.6	9.5	16.1	14.8	4.4	1.3	0.4
	Beef muscle 1	5.6	7.3	10.1	16.8	17.2	5.2	1.6	0.5
	Beef muscle 2	5.7	7.5	10.5	17.4	17.8	5.6	1.8	0.6
	Porcine skin	5.2	6.9	9.7	16.4	15.6	4.7	1.4	0.4
	Beef fat	5.1	7.1	10.6	18.5	15.4	5.6	2.0	0.7
	Beef tendon	5.2	7.2	10.6	18.5	15.5	5.6	2.0	0.7
K-laser	Chicken muscle	6.9	9.1	12.9	21.7	21.1	8.5	3.4	1.4
	Porcine muscle	4.6	6.1	8.8	14.9	13.5	3.6	1.0	0.3
	Beef muscle 1	5.8	7.6	10.8	18.1	17.7	5.9	2.0	0.7
	Beef muscle 2	5.5	7.2	10.1	16.8	17.0	5.1	1.5	0.5
	Porcine skin	4.9	6.6	9.4	16.1	14.7	4.4	1.3	0.4
	Beef fat	5.1	7.1	10.4	18.2	15.3	5.4	1.9	0.7
	Beef tendon	4.9	6.8	10.1	17.7	14.7	5.1	1.8	0.6

## Data Availability

The datasets used and analyzed during this study are available from the corresponding author on reasonable request.

## Supplementary Materials

The following supporting information can be downloaded at: https://www.mdpi.com/article/10.3390/biomedicines10123204/s1, Table S1: Details about the tissue specimens used for penetration depth measurements; Figure S1: Power of the EMS laser and the K-laser with its different modes measured for a time period of 10 min; Figure S2: Light intensity in time for the K-laser’s ISP mode at different set powers; Figure S3: Standard deviations of the residuals after computing best-fitting curves for the penetration depth curves for different tissues.
