# Supplementary material for: More than Ninety Percent of the Light Energy Emitted by Near-Infrared Laser Therapy Devices Used to Treat Musculoskeletal Disorders Is Absorbed within the First Ten Millimeters of Biological Tissue
Source: Biomedicines. 2022 Dec 9;10(12):3204. doi: 10.3390/biomedicines10123204 (PMC9775104; doi:10.3390/biomedicines10123204)
Supplement: Supplementary file 1 [file biomedicines-10-03204-s001.zip › LaserPaper Supplement.pdf]

biomedicines

More than ninety percent of the light energy emitted by near-infrared laser therapy devices used to treat musculoskeletal disorders is absorbed within the first ten millimeters of biolog-ical tissue

Supplementary Material

Table S1. Details about the biological tissue specimens used for penetration depth measurements.

Tissue	Chicken Muscle	Porcine Muscle	Beef Muscle	Beef Muscle	Porcine Skin	Beef Fat	Beef Ten-don	Porcine Bone
Number of sam-ples	12	15	10	9	9	6	3	6
Minimum thick-ness [mm]	1.90	1.60	2.35	2.45	4.20	2.00	2.55	14.0
Maximum thick-ness [mm]	15.0	14.0	24.0	21.0	21.0	16.5	13.0	16.0

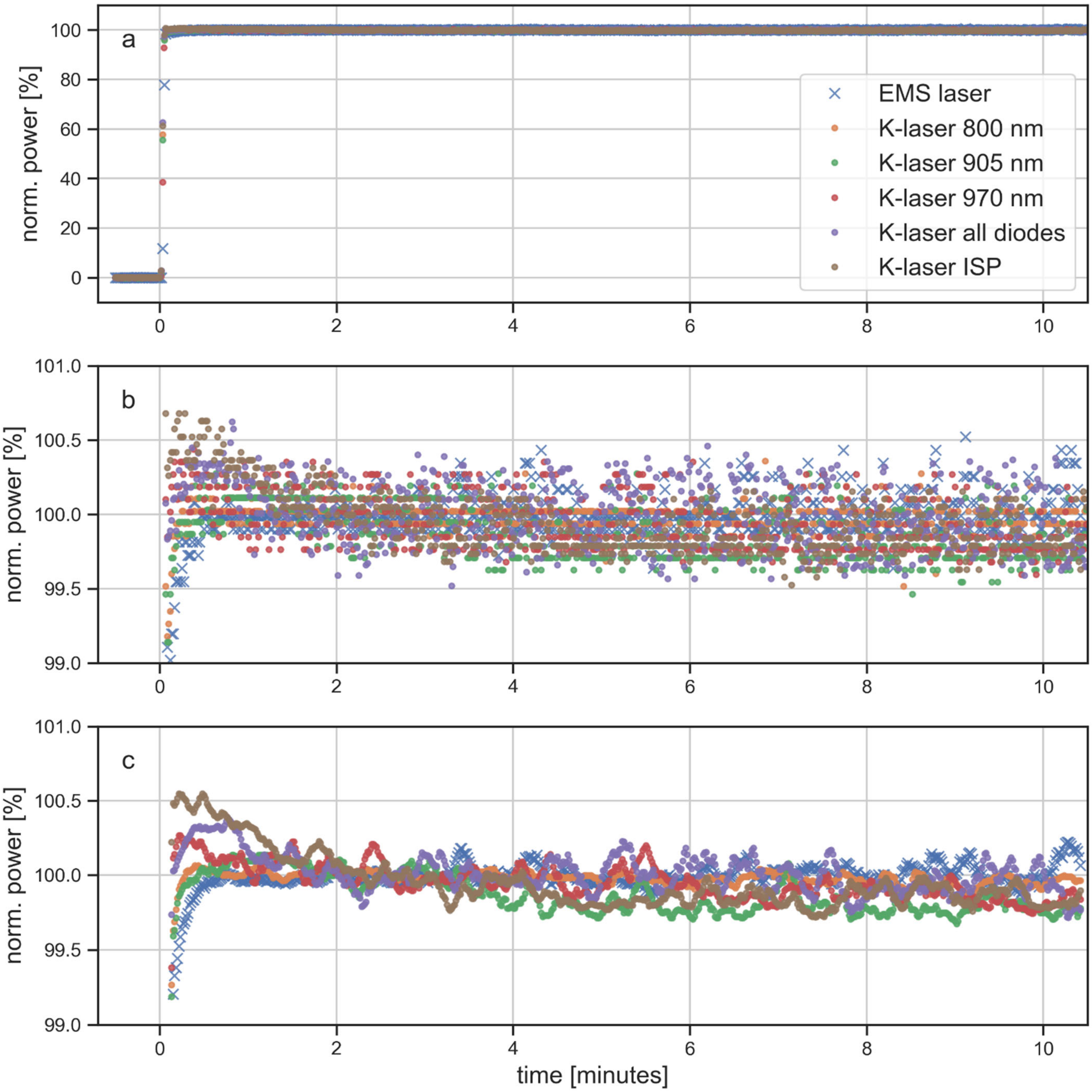

Figure S1. Power of the EMS laser and the K-laser with its different modes measured for a time period of 10 minutes. The power measurements of each signal were normalized to the mean value between minute two and minute 4. The same signals were plotted showing the full range (**a**) and showing the range around 100% in detail (**b, c**). The signals were recorded with a power sensor that operated with a sampling rate of 1 Hz. The signals are plotted directly (**a, b**) as well as after applying a moving-average filter with a window length of 10 samples (**c**). It can be seen that after the laser therapy devices were switched on, the power remained stable within 1% for the recorded ten minutes. The K-laser operated in ISP mode showed a minor initial overshoot of 0.7%, but stabilized after two minutes.

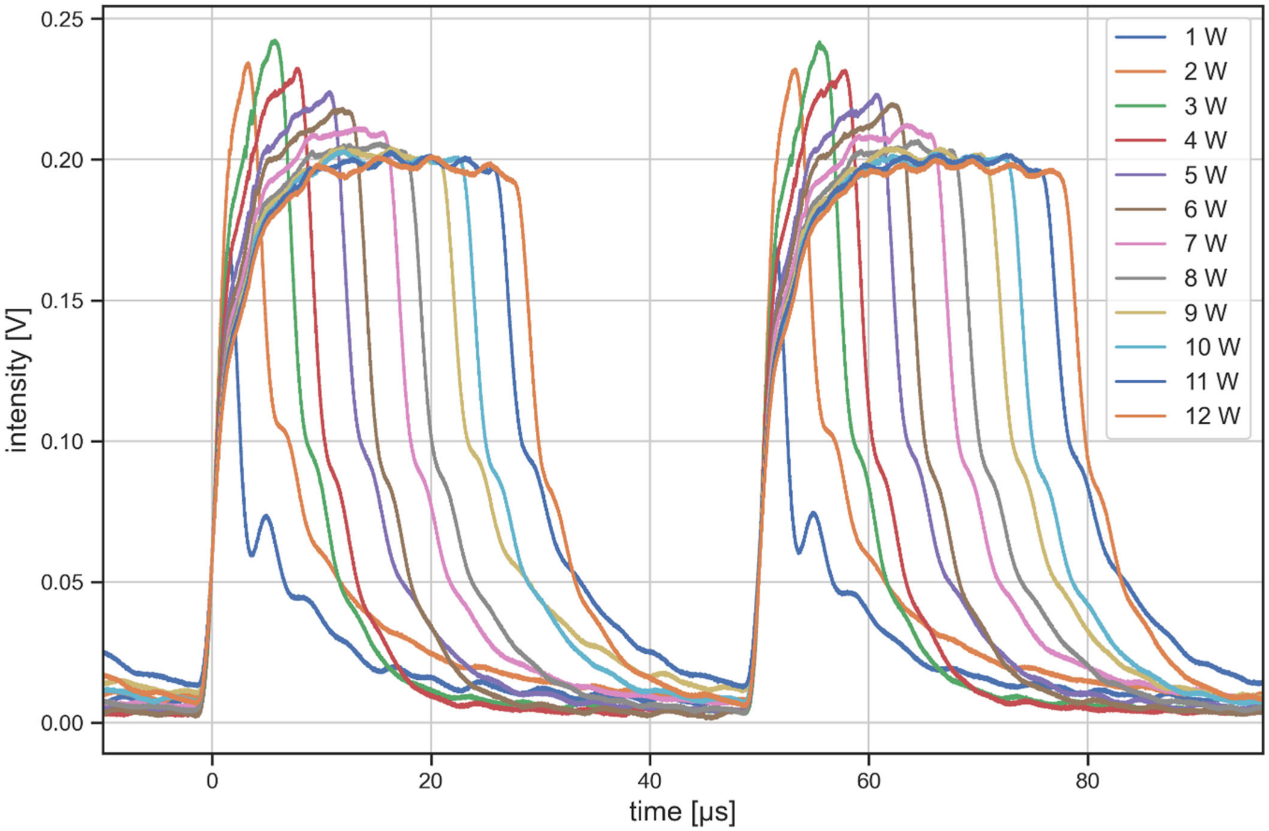

Figure S2. Light intensity in time for the K-laser's ISP mode at different set powers. The intensity was recorded using the fast photodiode connected to the oscilloscope, which recorded the signals in Volts.

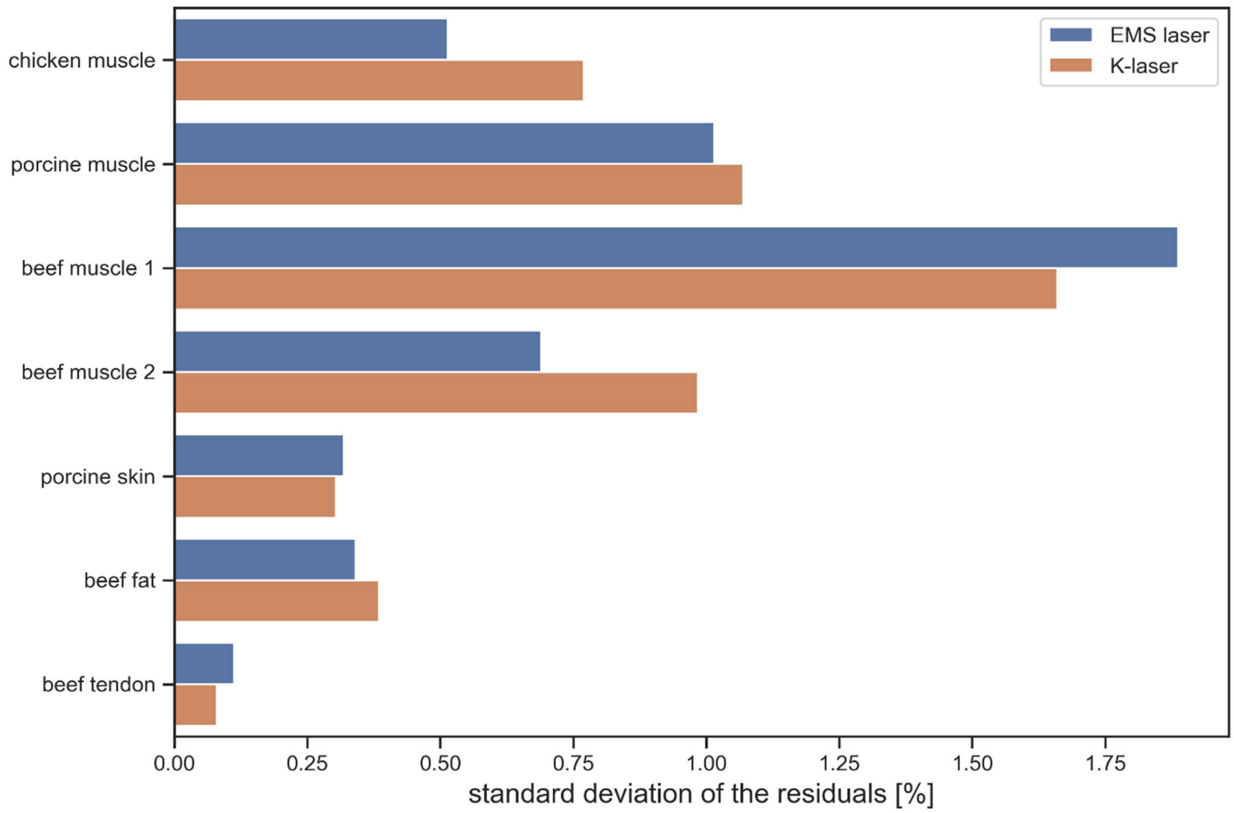

Figure S3. Standard deviations of the residuals after computing best-fitting curves for the penetration depth curves for different tissues.
